# Development and Biomechanical Study of a New Open Dynamic Anterior Cervical Nail Plate System

**DOI:** 10.1111/os.12622

**Published:** 2020-02-19

**Authors:** Xiao‐feng Zhao, Yi‐bo Zhao, Xiang‐dong Lu, Wen‐xuan Wang, De‐tai Qi, Xu Yang, Xiao‐nan Wang, Run‐tian Zhou, Yuan‐zhang Jin, Bin Zhao

**Affiliations:** ^1^ Department of Orthopaedics The Second Hospital of Shanxi Medical University Taiyuan Shanxi China

**Keywords:** Anterior cervical spine plate, Biomechanics, New type

## Abstract

**Objective:**

To develop a new type of open dynamic cervical spine system and study its biomechanical properties.

**Methods:**

Ten fresh goat spine specimens were used in this study. Using high precision digital display grating displacement sensor system, a new type of open dynamic nail plate fixation was compared with Atlantis nail plate fixation in terms of the stability of cervical vertebrae, pull‐out strength, and fatigue strength.

**Results:**

Biomechanical tests showed: (i) the new type of open dynamic cervical anterior nail plate system has similar three‐dimensional stability as the Atlantis nail plate system, and can ensure the stability of anterior cervical fixation surgery; (ii) the fatigue life and fatigue strength of this new open dynamic anterior cervical nail plate and Atlantis nail plate are similar, and can adequately maintain the cervical stability after anterior bone graft fusion, to ensure long‐term safety and efficacy of the nail plate system in the body; and (iii) the overall fixed performance of the new type of open dynamic cervical nail system is satisfactory.

**Conclusion:**

The new type of open dynamic anterior cervical nail plate system has satisfactory biomechanical characteristics and cervical spine stability effect.

## Introduction

Cervical spondylosis is a degenerative disease of the cervical vertebrae. Based on this, pathological changes such as nucleus pulposus prominence, intervertebral joint hyperplasia, and ligament hypertrophy which causes compression or stimulation of the spinal cord resulting in dysfunction and even loss of feeling, movement, reflexes and defecation. In recent years, with the changes in people's lifestyles and work patterns, the incidence of cervical spondylosis has quietly increased. The trend has become a serious threat to the health of middle‐aged and elderly people. Due to the characteristics of insidious onset and complicated complications, many patients with cervical spondylotic myelopathy have been in a more serious clinical state at the time of diagnosis. Therefore, for the patients with this disease, surgical treatment is the most direct and effective form of therapy. The anterior cervical surgery has smaller surgical trauma. In the meantime, it can directly remove the diseased intervertebral disc and the osteophyte behind the vertebral body. The direct decompression can relieve the compression of the posterior spinal cord and achieve a complete decompression effect; at the same time, it can ensure good bone fusion of the vertebral body so that stability of the cervical vertebra is ensured.

Ever since Robinson and Smith^1^ used anterior cervical surgery to treat cervical spondylopathy for the first time in the 1960s, this clinical practice has proven to be the most effective clinical treatment for cervical spine‐related diseases. This surgical method can directly decompress the spinal cord in front of the degenerative intervertebral discs, and provide firm fixation through cage or titanium mesh bone graft with anterior cervical internal fixation system to maintain postoperative stability of the cervical spine and cervical lordosis. However, there are many limitations in the current design and operation of the traditional cervical plate. Especially for patients with osteoporosis, there is a higher risk of spine plate displacement, screw loosening, and higher repair rate of the internal fixation plate. Robinson^2^ and Smith^3^ proposed anterior cervical discectomy fusion (ACDF) treatment of cervical spondylosis. This procedure directly relieved the compression of the spinal cord. The symptoms of the patient were greatly improved. With the advancement of clinical practice, the anterior cervical decompression and bone graft fusion surgery method has rapidly developed and become a classic surgical treatment of cervical disease. Subsequently, various cervical anterior plate systems were developed with this technique, including: bicortical screws (cortical bone screws), non‐locking non‐robust; single cortical screws (cancellous bone screws), locked sturdy; cortical screws (rotatable cancellous bone screws), angled half‐restricted; and single cortical screws (slip cancellous bone screws), slip semi‐limited^4^. However, the traditional cervical anterior plate has certain limitations in the design and operation of the plate itself. The Caspar cervical anterior plate is fixed with a bicortical screw. When it is fixed, it needs to penetrate the cortical bone of the posterior edge of the cervical vertebra to increase the fluoroscopy time. The screw and steel plate of the cervical anterior plate of the CSLP are fixed. During the plating process of the traditional cervical anterior plate, the titanium plate will slide in the anterior direction, making it difficult to accurately control the entry point, so that the titanium plate cannot achieve the expansion pressure required for surgery to extend the operation time. When the nail hole is used as the titanium plate screw to penetrate the nail hole, it is necessary to remove the nail, which will increase the amount of bleeding, making it difficult to expose, and increasing the difficulty of operation.

In this study, a new type of open dynamic cervical anterior nail plate was designed and developed to compensate for the technical problems in the clinical operation of the cervical anterior nail plate system, and was subjected to biomechanical studies, such as stability test and fatigue test, to explore the advantages of this system. The aims were: (i) design the experiment of three‐dimensional motion stability, fatigue, and vertical extraction to test the biomechanical properties of the new open dynamic anterior cervical nail plate system; (ii) summarize the design advantages of the new type of open dynamic nail plate system; and (iii) analyze the biomechanical properties of the new type of open dynamic nail plate system.

## Materials and Methods

### *Composition and Related Parameters of a New Type of Open Dynamic Cervical Anterior Nail Plate System*

**Figure 1:**
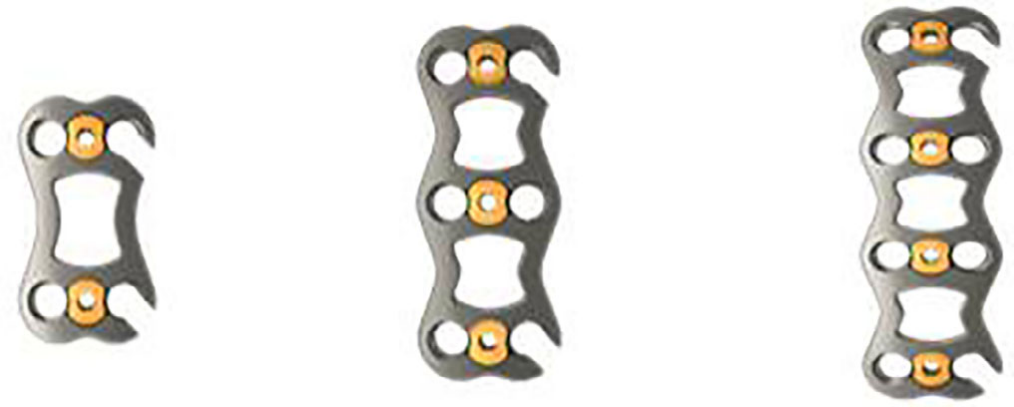
Various models of open‐power cervical anterior titanium plate.

**Figure 2:**
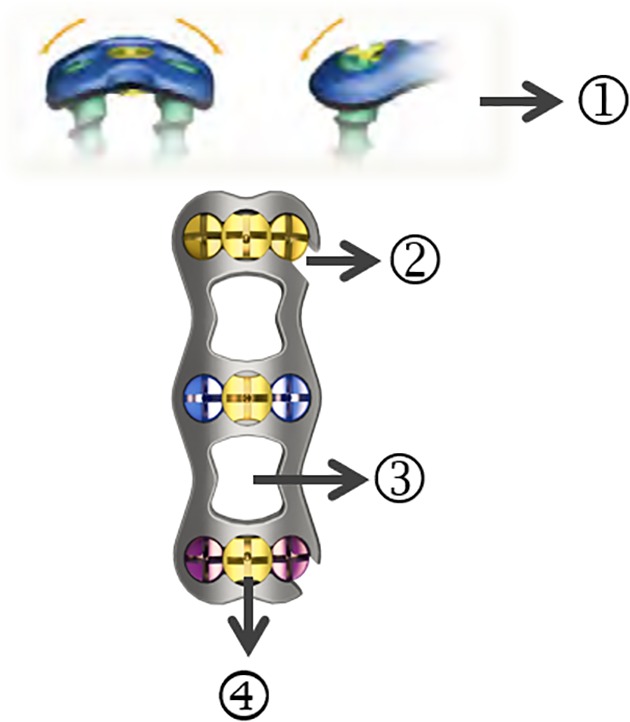
The advantage of open‐power cervical anterior titanium plate: ① Slope edge and smooth surface reduce soft tissue irritation. Pre‐bent steel plate reduce the use of the bender. ② The nail hole on one side of the titanium plate is “C” shaped and is not completely closed. ③ Large perspective window for easy Intraoperative and postoperative observation, convenient bone grafting. ④ Locking shrapnel to prevent the screw from automatically exiting.

**Figure 3:**
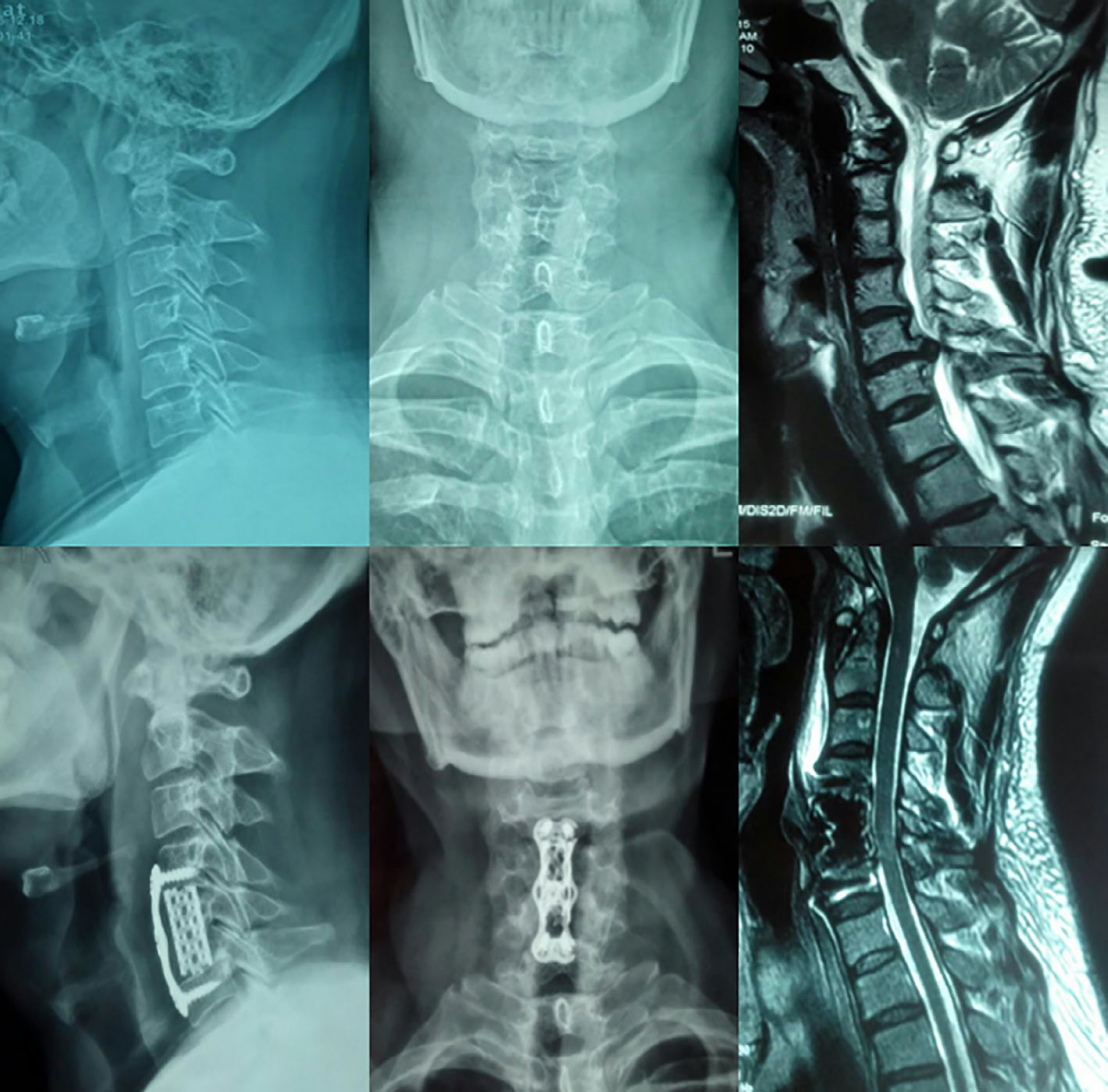
The application of the new open dynamic anterior cervical nail plate system in anterior cervical decompression surgery.

**Figure 4:**
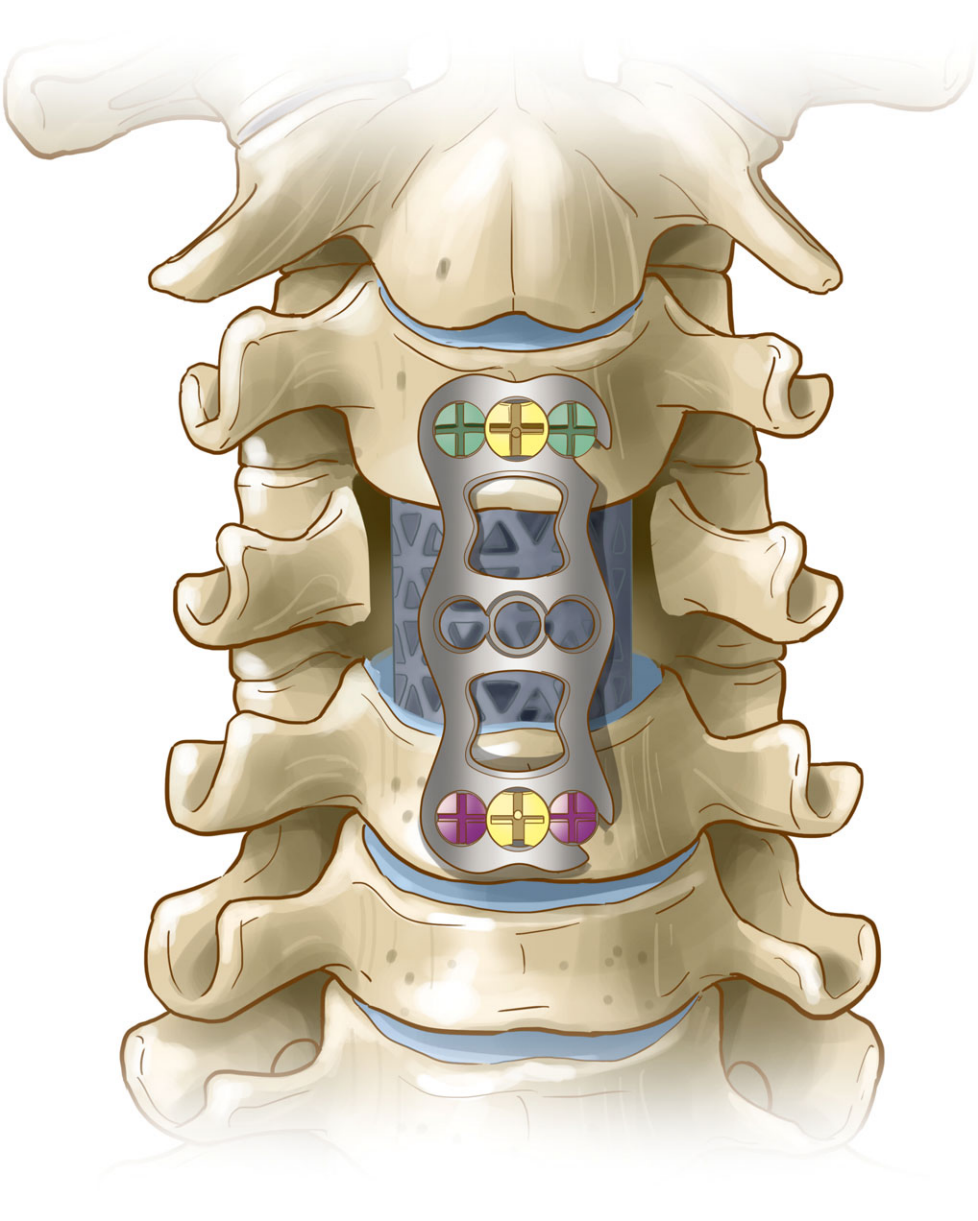
The working principle of the new open dynamic anterior cervical nail plate system (coronal plane).

**Figure 5:**
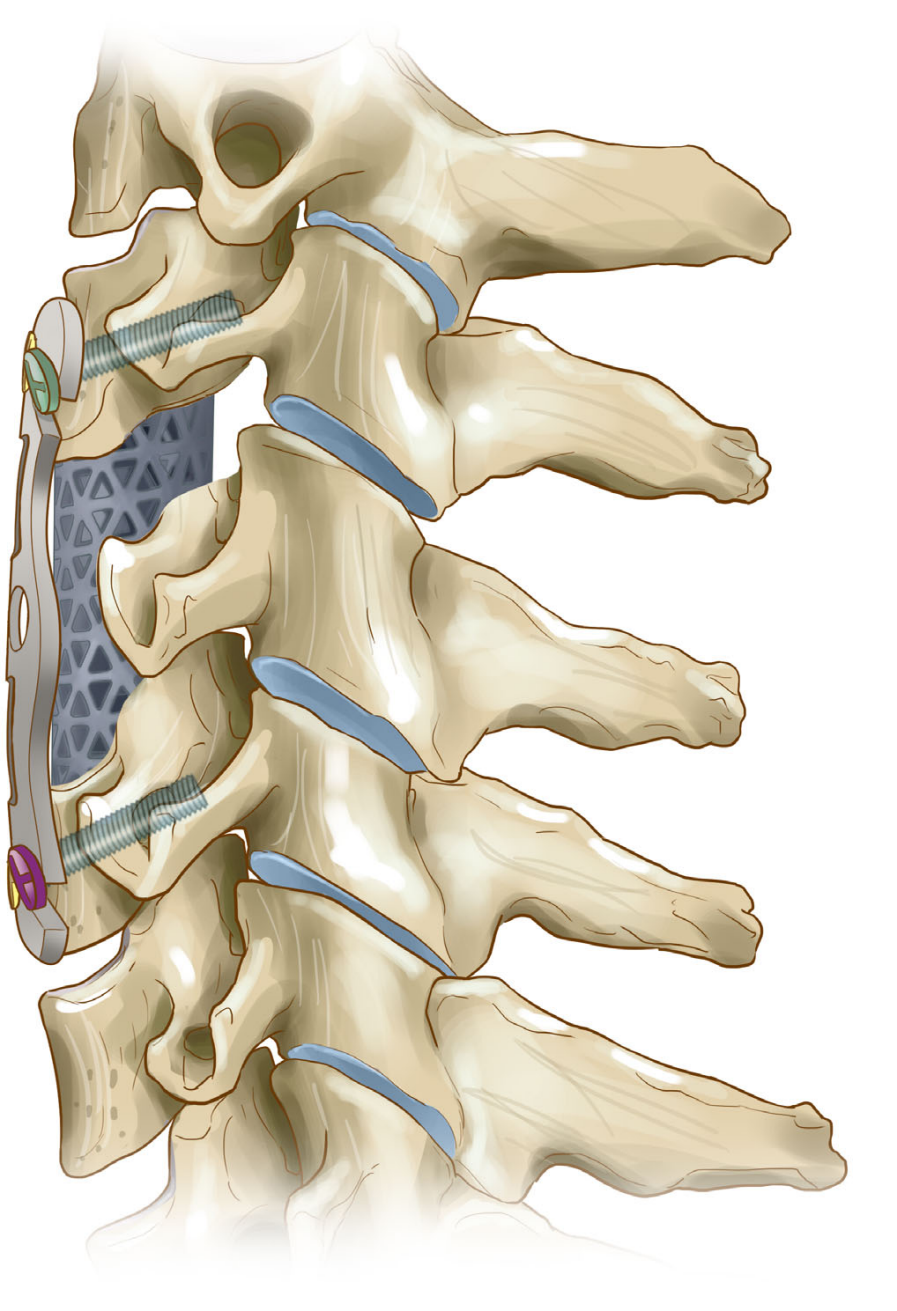
The working principle of the new open dynamic anterior cervical nail plate system (sagittal plane).

The new cervical spine anterior fixation system is made of medical titanium alloy. Its components include steel plates, fixing screws, and locked plates. The steel plate has a width of 17 mm, length of 22–65 mm, increment of 3 mm, and thickness of 2.2 mm. Its upper and lower ends are curved toward the cervical side, two screw holes are provided at both ends, and the screw hole on one side is “C” shaped and is not entirely closed. The middle of the screw hole has a locked plate, and a large perspective window is set at the center of the steel plate. Screw length range is 10–20 mm, diameter 4 mm. The screw design is a self‐tapping screw with two distinguishing colors, fixed angle, and adjustable angle (Figs 1, 2, 3, 4, 5).

## Biomechanical Test

### *Stability Test*

#### *General Information*

Ten fresh cervical specimens (C_1_–C_7_) were prepared from healthy goats aged 12–24 months (mean 16 months). X‐ray, computed tomography (CT), and magnetic resonance imaging (MRI) were performed on each specimen to exclude cervical and tumor lesions. The two‐layer preservation plastic wrap was used to wrap the specimens and stored at −20°C for cryopreservation. Before the experiment, the specimens were thawed at room temperature in fixed humidity for 8–10 h. The soft tissues such as muscles and fat around the vertebral bodies were removed, and the ligaments, bone structure, and intervertebral discs were preserved. The jet tray powder was prepared and used to embed and fix the ends of the vertebral bodies of the C_1_ and C_7_ specimens, respectively, on a self‐made aluminum plate of 10 cm × 10 cm size to ensure that the specimens were maintained in a stable and fixed state. During the experiment, the test pieces were periodically sprayed with 0.9% saline to maintain the humidity of the specimen.

#### *Experimental Grouping and Surgical Methods*

Ten goat cervical spine specimens were randomly divided into two groups, A and B, with five specimens in each group. The new anterior cervical decompression, bone graft fusion, and open dynamic nail plate fixation surgery were performed on group A specimens, while anterior cervical decompression, bone graft fusion, and Atlantis screw plate^5^ internal fixation surgery were performed on group B specimens. Then, the C_5_ vertebral body and the adjacent intervertebral disc were removed by surgery, and the defect segment was carefully trimmed. The appropriate size of the autologous iliac bone was selected for the implanted defect site. Afterward, an anterior cervical plate of appropriate length was selected to cover the bone graft, and the nail plate was firmly fixed on the C_4_ and C_6_ vertebral bodies.

#### *Three‐Dimensional Motion Stability Test*

Both groups of test specimens underwent the three‐dimensional motion stability test using high‐precision digital grating displacement sensors^6^, ^7^. First, the normal specimens of both groups were fixedly installed using the MTS858 biomechanics experimental machine, and the sensors were connected. After the specimen became stable, a torque load of 150 N was applied to the specimen from the head end, and the torque was 2.0 Nm, allowing the spine to move in six directions within the physiological range, recording angular displacement changes^8^. If the angular displacement in any one direction increased significantly, it indicated that the segment was unstable; if the angular displacement in any one direction decreased significantly, it indicated that the segment was stable^9^. After the surgery, the same test was conducted on both A and B groups. Before each data acquisition, the specimen was loaded/unloaded for three cycles to eliminate the influence of viscoelasticity of the specimen on the experimental data. The data was recorded after the third load was applied for 30 s.

### *Fatigue Test*

**Figure 6:**
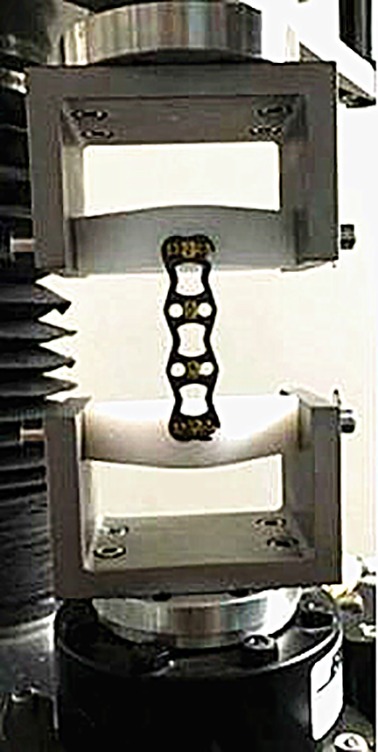
Fatigue experiment of open‐power cervical anterior titanium plate.

Two sets of the new open dynamic nail plate and two sets of Atlantis nail plates were fixed on the MTS 858 experimental biomechanics machine. According to ASTM F1717‐15 standard, sinusoidal waves were used in high‐frequency dynamic bending fatigue tests on steel plates, and both sides of the steel plates were fixed to two pieces of ultra‐high‐molecular‐weight polyethylene (UHMWPE). The load was 100 N, loading frequency was 0.5 Hz, the number of cycles was 106, and the load ratio was 10^10^. The data were recorded every 2000 cycles until the plate bolts broke or reached 106, then the maximum load, cycle times, and fracture sites were recorded. If the steel plate had no fracture after the experiment was completed, the loosening of the steel plate screws was observed. Fatigue (σ‐1) was calculated using the formula σmax = α × σ‐1 to calculate the maximum stress at the edge of the titanium plate screw hole, where α was the stress concentration factor^11^ (Fig. 6).

### *Vertical Extraction Test*

**Figure 7:**
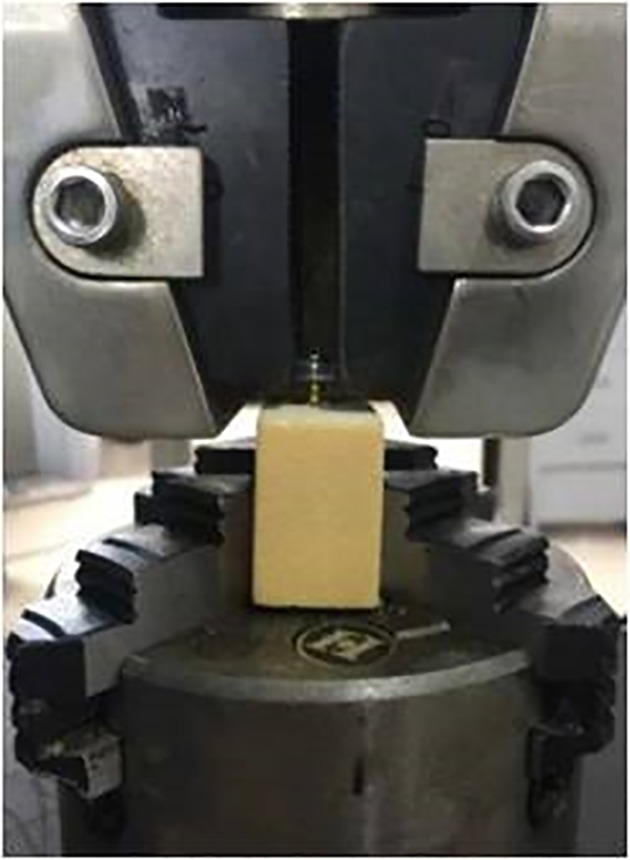
Pull out the experiment of open‐power cervical anterior titanium plate.

Two sets of the new open dynamic nail plates and two sets of Atlantis nail plates were fixed on the CMT5105 electronic universal testing machine. According to ASTM F543‐13e1 standard, the steel plates were fixed to a 150 mm long, 50 mm wide, and 30 mm high simulated cancellous bone made of polyurethane. The steel plate screw system was under vertical extraction test, and the maximum pull‐out force was recorded (Fig. 7).

## Statistical Analysis

SPSS 21.0 software was used for analysis of the measured data. The measurement data were all expressed in (±). For both groups, the paired *t*‐test was used to compare the normal state and the state after the nail plate system was fixed, as well as the range of cervical motion and the neutral zone. The *t*‐tests of the two independent samples were compared between the normal state and the state after the posterior cervical spine fixed by different anterior screw systems in both groups or the range of motion and neutral area. The comparison of different nail plates in the fatigue test and pull‐out force test was performed using *t*‐tests with two independent samples, with a significant level of 0.05.

## Results

### *ROM and Neutral Zone*

**Figure 8:**
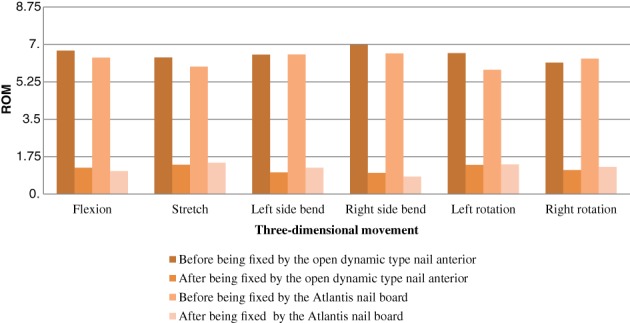
ROM diagram of the two groups before and after fixation of the fixed cervical spine plate.

There was no significant difference between the two sets of cervical vertebrae specimens in the ROM and neutral zone before nail plate fixation (*P* > 0.05), and there was no significant difference between ROM and neutral zone after nail plate fixation surgery (*P* > 0.05). There was a statistically significant difference between ROM and neutral zone measured after surgery in different direction of movement (*P* < 0.05) (Tables 1, 2 and Fig. 8).

**Table 1:** ROM (°) of two groups of cervical C_4_–C_6_ segments in two states

Direction of movement	Group A (*n* = 5)		Group B (*n* = 5)	
	Normal	After being fixed	Normal	After being fixed
Flexion	6.71 ± 0.93	1.23 ± 0.16*	6.38 ± 0.84	1.07 ± 0.26*
Stretch	6.39 ± 0.89	1.37 ± 0.19*	5.96 ± 0.91	1.46 ± 0.57*
Left side bend	6.52 ± 1.14	1.01 ± 0.21*	6.53 ± 1.03	1.23 ± 0.38*
Right side bend	6.98 ± 1.07	0.99 ± 0.19*	6.58 ± 1.29	0.82 ± 0.21*
Left rotation	6.59 ± 0.97	1.36 ± 0.34*	5.81 ± 0.72	1.39 ± 0.48*
Right rotation	6.15 ± 0.94	1.12 ± 0.09*	6.33 ± 1.16	1.27 ± 0.14*

* Compared with the normal state after fixation, *P* < 0.05.

**Table 2:** Pull‐out experimental force (N) of the two types of nail board systems (mean±SD)

Direction of movement	Group A (*n* = 5)		Group B (*n* = 5)	
	Normal	After being fixed	Normal	After being fixed
Flexion position	5.73 ± 1.29	0.96 ± 0.29*	6.02 ± 0.91	0.91 ± 0.43*
Side bend	5.29 ± 0.77	0.79 ± 0.14*	5.27 ± 1.06	0.87 ± 0.32*
Rotation bit	5.12 ± 0.63	1.02 ± 0.43*	5.11 ± 0.59	0.94 ± 0.27*

* Compared with the normal state after fixation, *P* < 0.05.

### *Fatigue Experiments and Pull‐Out Experimental Force*

**Figure 9:**
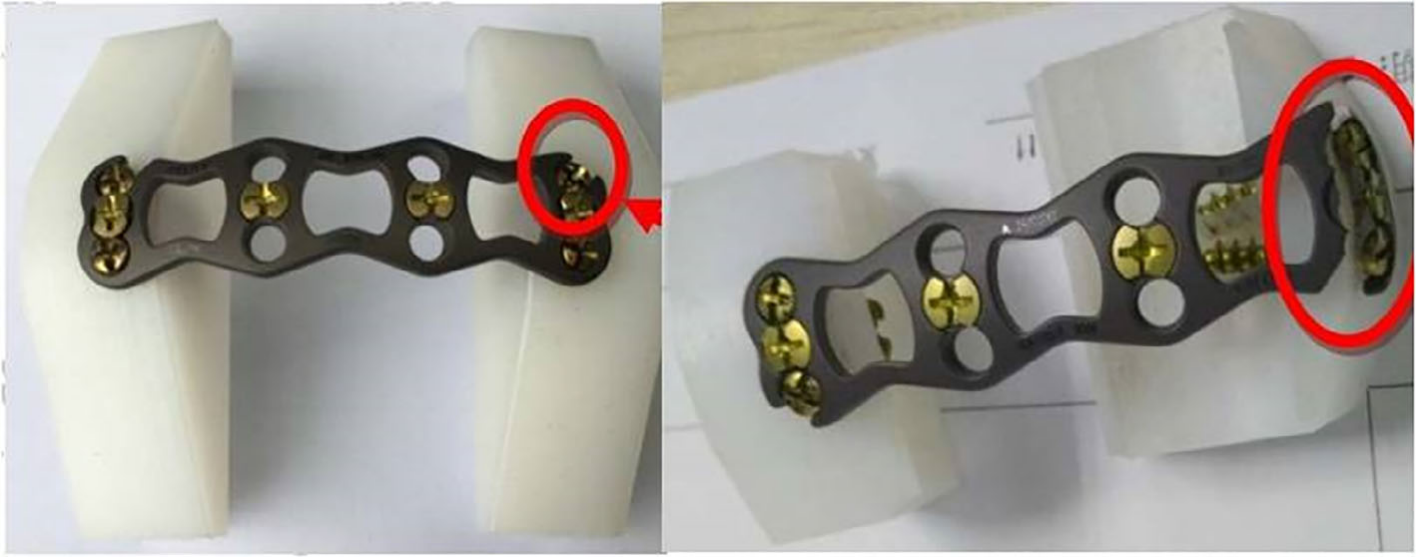
Fracture diagram of fatigue test steel plate.

The cycle time of the new open dynamic nail plate was 6.0 × 10^5^ on an average, the strength of the nail plate was 503.31 MPa, the maximum stress at the edge of the nail hole was 644.24 MPa. The average cycle time of the Atlantis nail plate was 6.1 × 10^5^, the board strength was 511.79 MPa, and the maximum stress at the nail hole edge was 655.09 MPa (Table 3). The results of pull‐out experiments showed no significant difference in the maximum pull‐out force between the two types of nail plate systems (*P* > 0.05) (Table 4 and Fig. 9).

**Table 3:** Fatigue experiments on the two nail board systems

Nails	Load (N)	Frequency (HZ)	Cycles	Fatigue strength (MPa)	Result
Open dynamic type nail anterior cervical plate	100	0.5	6.0 × 10^5^	503.31	Damage
Atlantis nail board	100	0.5	6.1 × 10^5^	511.79	Damage

**Table 4:** Pull‐out experimental force (N) of the two types of nail board systems (mean±SD)

Test index	Nail board system		Simple screw	
	Open dynamic type nail anterior cervical plate	Atlantis nail board	Open dynamic type nail anterior cervical plate	Atlantis nail board
Maximum pullout force	423.69 ± 27.18	397.52 ± 23.64	281.43 ± 23.11	268.86 ± 19.63

## Discussion

### *Design Advantages of the New Type of Open Dynamic Nail Plate System*

The new type of the open dynamic nail plate system is made of medical titanium alloy. The titanium alloy has lightweight, good biocompatibility, and high corrosion resistance. It is also non‐magnetic and does not affect the follow‐up of patients after MRI examination^12^. The nail holes at both ends of the titanium plate are “C” shaped and not entirely closed. During the process of installation, it is not necessary to remove the pegs. The open side of the titanium plate is directly clamped into the unique peg opener and is set nails under stable pressure, which ensure the pressure to open effect and also facilitate the operation. There are locked plates at both ends of the titanium plate to facilitate the operation, which effectively prevent loosening of the screws, and increase the safety of the operation. Both ends of the titanium plate are designed with a sloped edge and a smooth surface to reduce the stimulation of the surrounding soft tissue. The titanium plate has a pre‐curved design, which not only reduces the use of the plate bender but also increases the conformation of the titanium plate to the bone surface, thereby efficiently avoiding uneven stress. The middle part of the titanium plate has a large perspective window to facilitate observation and intraoperative bone grafting. The screws are designed as self‐tapping screws, which can be divided into fixed angles and adjustable angles. The colors of the screws can be distinguished, which not only reduces the use of wiretapping but also quickly discerns the diameter and type.

### *Biomechanical Properties of the New Type of Open Dynamic Nail Plate System*

Biomechanical evaluation of spinal instrumentation includes strength tests, fatigue tests, and stability tests^13^. The experimental results of three‐dimensional motion stability test of the cervical spine showed that the new open dynamic nail plate system could achieve the same three‐dimensional stabilization effect as the Atlantis nail plate system after fixation, indicating that the new open dynamic nail plate system is practical and effective in anterior cervical spine surgery. Fatigue test results showed that the fatigue life and fatigue strength of the two nail plates were similar. The new type of open dynamic nail plate system could withstand an average load of 6.0 × 105 times before fracture, and its fatigue strength was 503.31 MPa. This fatigue strength was sufficient to maintain the stability of the cervical spine after anterior surgery, and the nail plate system was safe and effective in the body for long‐term usage. The results of the pull‐out experiment showed no significant difference in maximum pull‐out force between the two types of nail plate systems irrespective of whether it was a simple screw or the entire nail plate system, indicating that the new open dynamic nail plate system had similar fixing effect and satisfactory fixing locking performance as compared to the Atlantis nail plate system.

### *Study Limitations*

The research on the biomechanical mechanism of cervical spine mainly includes animals (*in vitro* and *in vivo*), human cadaver specimens, and computer simulation experiments (finite element analysis method). Due to the difficulty in obtaining specimens and the high cost, human cadaver specimens were not used in this study. The goat cervical spine is known to be the most suitable model for simulating human cervical spine. Therefore, goat cervical spine specimens were used for biomechanical tests in this study. However, the biomechanical effects of muscle tissue and the synergistic effects of soft tissues such as ligaments have a major effect in maintaining spinal stability^14^, ^15^. Therefore, we need to further test the nail plate system on intact human cadavers. Biomechanical tests establish a three‐dimensional finite element model of the new open dynamic nail plate system in order to reflect the stress distribution of the titanium plate. The fatigue test was performed on a simulated vertebral body resection model. There was no bone graft support in this model and no cervical composite load, resulting in some differences with the *in vivo* situation.

In summary, the new open dynamic nail plate system has satisfactory biomechanical properties and can provide an effective stabilizing effect on the cervical spine.

## Acknowledgments

This work was supported by Shanxi Soft Science Research Project (2018041027‐2).

There were no relevant financial activities conducted for this study outside the submitted work.
